# An Evaluation Model for the Quality of Frying Oil Using Key Aldehyde Detected by HS-GC/MS

**DOI:** 10.3390/foods11162413

**Published:** 2022-08-11

**Authors:** Xiaofang Liu, Shuo Wang, Shigeru Tamogami, Jieyu Chen, Han Zhang

**Affiliations:** 1 Faculty of Bioresource Sciences, Akita Prefectural University, Akita 010-0195, Japan; 2 School of Tourism and Cuisine, Yangzhou University, Yangzhou 225127, China; 3 Key Laboratory of Chinese Cuisine Intangible Cultural Heritage Technology Inheritance, Ministry of Culture and Tourism, Yangzhou 225127, China; 4 School of Food Science and Engineering, Yangzhou University, Yangzhou 225127, China

**Keywords:** headspace-gas chromatography/mass spectrometry, pentanal, frying oil, carbonyl value, quality evaluation

## Abstract

To establish a practical model for evaluating the oxidation of frying oil using aldehydes, the aldehydes of 10 commercial oils during frying at 180 °C were identified using headspace-gas chromatography/mass spectrometry, and the changes of common aldehydes and their correlation with carbonyl values (CV) were analyzed. The results showed that the total peak area of aldehydes increased significantly with heating time, which was related to the fatty acid and tocopherol contents of the oils. There were four common aldehydes with different trends during frying, namely, pentanal, hexanal, (*E*)-hept-2-enal, and nonanal. Moreover, pentanal with a high correlation with CV was selected as the quality evaluating index of frying oil due to its stable accumulation over time. Based on the linear fitting relationships between CV and pentanal, as well as the initial content ratio of linoleic acid to palmitic acid and total tocopherols in oils, a predictive model was established for evaluating the quality of frying oils with high precision and non-reagent by using mass spectrometry. In summary, this work provides theoretical support for using aldehyde as the quality evaluation index of frying oil and provides a new idea for evaluating oil deterioration from the perspective of volatile compounds.

## 1. Introduction

Deep frying is a popular method of food preparation throughout the world, because it is not only simple and fast but also imparts a unique flavor to fried foods. French fries are the most popular fried foods around the world, with a $30–35 billion annual chips market worldwide [1]. During frying, many chemical reactions, such as oxidation, hydrolysis, and decomposition, occur in the hot oil, producing a series of volatile compounds, including aldehydes, alcohols, hydrocarbons, acids, and heterocyclic compounds [2,3]. The most abundant volatile compounds produced are aldehydes, mainly formed by the thermal oxidation of unsaturated fatty acids linked with triacylglycerol during frying [4], and aldehydes are considered to be ubiquitous in frying oil. The generated aldehydes could impart undesirable flavors to the oil, such as oily, green, paint, metallic, and beany [5], which in turn affect the oil quality. Consequently, there is a growing body of literature that recognizes the importance of aldehydes in frying oil [6,7,8].

The thermal oxidation of oil during frying is an important reason for oil deterioration. Traditionally, deterioration indicators such as the carbonyl value (CV) have been used to evaluate the thermal oxidation of frying oils. CV refers to the content of carbonyl compounds such as aldehydes and ketones in the oxidation products and reflects the deterioration degree of frying oil [9]. Unfortunately, CV measurement requires chemical reagents, which are not environment-friendly. So far, it remains an urgent challenge to develop an alternative method for the determination of CV in a safer and simpler way. For this problem, the potential of volatile compounds, especially aldehydes, has recently attracted more and more attention due to their high correlation with the deterioration process of frying oil. Correspondingly, some aldehydes, such as (*E*,*E*)-2,4-decadienal [10], (*E*,*E*)-2,4-heptadienal, (*E*)-2-decenal [11], and (*E*)-2-undecenal [12], have been suggested to show the possibility of being an evaluation index for evaluating the quality of frying oil. However, no specific evaluation model has been established for investigating the relationships between oil deterioration and aldehyde generation. The fact that could be attributed to the diversity of frying conditions (temperature, time, type of oil, and food) leads to the complexity and diversity of the generation of volatile compounds, which increases the difficulty of using aldehydes as a quality evaluation index of frying oil and challenges its applicability and reliability. Since the generation and change of aldehydes are related to the quality of frying oil, there is still an urgent need to use aldehyde as an evaluation index of oil deterioration to explain the quality change of frying oil from the perspective of flavor chemistry.

The present study aimed to determine the potentially important aldehydes in 10 commercial edible oils during frying and to establish a feasible evaluation model to replace the complex traditional measurement method of CV. Firstly, the initial composition of 10 oils was determined to study the effect on the generation and change of volatile compounds. The aldehydes in 10 edible oils during frying were identified by using headspace-gas chromatography/mass spectrometry (HS-GC/MS). Furthermore, the correlation between the changes of common aldehydes and CV during frying was revealed. Finally, a predictive model for evaluating the deterioration of frying oil was established based on the peak area of the pentanal. In this study, the volatile compounds and deterioration of frying oil were linked, providing a new perspective for evaluating the deterioration of oils.

## 2. Materials and Methods

### 2.1. Preparation of Frying Oil Samples

The commercial edible oils selected for the present study were six refined oils (olive oil (OL), safflower oil (SF), rapeseed oil (RS), rice bran oil (RB), corn oil (CO), and soybean oil (SB)), two roasted oils (sesame oil (SS) and perilla oil (PL)), and two natural pressed oils (natural sesame oil (NS) and natural perilla oil (NP)). All 10 oils and frozen par-fried French fries were bought in local marketplaces (Akita, Japan).

The frying process was carried out by using four liters of each oil to fry the French fries in a restaurant-style stainless steel electric fryer (TF-40A, Taiji & Company Limited, Kanagawa, Japan) at 180 °C. During frying, the temperature of the frying oil was kept constant at 180 °C with 100 g of French fries being fried for 3 min at 27-min intervals. This operation was repeated for 5 h each day over 5 consecutive days and 200 mL of frying oil was taken at the end of each day, with no new oil being added to the fryer. The oil samples were stored at −18 °C immediately before analysis.

### 2.2. Chemicals

Hexane, methanol, acetic acid, isopropanol, butylated hydroxytoluene, potassium hydroxide, 2,4-dinitrophenylhydrazine (2,4-DNPH), and butanol were purchased from FUJIFILM Wako Pure Chemical Corporation (Osaka, Japan). Hydrochloric acid was purchased from Kanto Chemical Company Limited (Tokyo, Japan). Standard reagents include fatty acid methyl standards, Vitamin E reference standards (d-α-, β-, γ-, and δ-tocopherol), 2,2,5,7,8-pentamethyl-6-hydroxychroman, and (*E*)-2-decenal were purchased from FUJIFILM Wako Pure Chemical Corporation. Custom alkanes blend standard of C6–C16 was provided by Restek Corporation (Bellefonte, PA, USA). Hexane and isopropanol were of high-performance liquid chromatography grade and all other chemicals were of analytical grade.

### 2.3. Determination of Oil Composition and Quality Index

The fatty acid composition of 10 oils was determined by gas chromatography (GC-2010, Shimadzu Company, Kyoto, Japan) using the method described in detail previously [13]. Briefly, fatty acid methyl ester was prepared by dissolving the oil sample (about 100 mg) in hexane (10 mL) and adding 0.1 mL methanol potassium hydroxide solution (11.2 g/100 mL) to vortex. An HP-88 capillary column (100 m × 0.25 mm i.d., 0.20 μm film thickness; Agilent Technologies International Japan, Tokyo, Japan) was used for separation. The temperature ramping program was increased from 120 °C to 170 °C at a rate of 10 °C/min, then at 4 °C/min increased to 250 °C, and held for 5 min. A flame ionization detector was used to detect samples with the detector temperature of 260 °C. The sample (1 μL) was injected at a split ratio of 1:30. All peaks were identified by comparison of their retention times with a standard fatty acid methyl ester mixture. Results are expressed in percent relative content.

The tocopherol content of 10 oils was investigated using high-performance liquid chromatography (Shimadzu, Kyoto, Japan). The sample (about 100 mg) was dissolved in a 10 mL volumetric flask with hexane and 100 μL of the internal standard 2,2,5,7,8-pentamethyl-6-hydroxychromium was added. The mobile phase consisted of hexane, isopropanol, and acetic acid (1000:6:5, *v*/*v*/*v*) and contained 5 μg/mL of butylated hydroxytoluene at a flow rate of 0.8 mL/min. A normal-phase Shodex silica 5Sil 4D analytical column (150 mm × 4.6 mm i.d., Showa Denko, Tokyo, Japan) was used for separation with the column oven was 40 °C. A fluorescence detector was performed for detection with an excitation wavelength of 298 nm and an emission wavelength of 325 nm. The concentration of each tocopherol was quantified using separate calibration curves prepared for α-, β-, γ-, and δ-tocopherol standards [14].

The CV of the oil samples was determined referring to the Japan Oil Chemists’ Society Official Method Tentative 13–2013 Carbonyl Value [15]. In brief, the oil sample (50–500 mg) was filled into 10 mL volumetric flasks with butanol. A 2,4-DNPH solution was prepared by dissolving 50 mg of 2,4-DNPH in 100 mL of butanol that contained 3.5 mL of concentrated hydrochloric acid. (*E*)-2-Decanal was dissolved in butanol to prepare standard solutions at concentrations of 100, 200, and 400 μmol/L. The standard solution or oil sample (1 mL) was mixed with 1 mL of 2,4-DNPH solution and placed in a 40 °C water bath for 20 min, then cooled to room temperature under tap water. Potassium hydroxide butanol solution (8 mL, 8 g/100 mL) was added to the above standard solution or oil sample and mixed evenly. After centrifugation at 3000 rpm for 5 min, the absorbance of samples and standards was detected with a spectrophotometer (benchmark plus microplate reader, bio rad, Tokyo, Japan) at 420 nm.

### 2.4. Determination of Aldehyde

Headspace (HS) analysis is a rapid volatile component extraction method that has been widely used [16,17]. The analysis of aldehydes in the headspace of the oil samples was performed using an HS-GC/MS system (QP2020, Shimadzu Company, Kyoto, Japan). For each trial, the oil sample of about 1 g was placed in a 20-mL vial which was sealed with a magnetic cap. The equilibrium was carried out at 80 °C for 30 min in an HS-20 headspace auto-sampler. After equilibrium, 1 μL of the sample was injected with a split ratio of 1:10. The carrier gas was helium at a constant flow of 11.0 mL/min. The column used was an SH-Rxi-5Sil MS capillary column (30 m × 0.25 mm i.d. × 0.25 μm film thickness, Shimadzu Company). The oven temperature was programmed to start from 40 °C, held for 5 min, and increased to 250 °C at a rate of 4 °C/min then kept for 3 min. The temperatures of the ion source and the interface were kept at 200 and 230 °C, respectively. The mass spectrometer was operated in full scan mode, and the mass spectra in the range of 35–350 *m/z* were recorded.

The identification of aldehydes was performed by matching the mass spectrum with the standard spectra provided by the National Institute of Standards and Technology (NIST17) database (Agilent Technologies Inc., Gaithersburg, MD, USA), and verified by matching their Kováts retention indices (RI) with the SH-Rxi-5Sil MS capillary column or DB-5 column that reported in the literature. The RI value of each volatile compound was calculated by using C6–C16 *n*-alkanes with the Van den Dool Equation [18]:

(1) $$\mathrm{RI}=100\times \left(n+\frac{{\mathrm{RT}}_{i}-{\mathrm{RT}}_{n}}{{\mathrm{RT}}_{n+1}-{\mathrm{RT}}_{n}}\right),$$

where RT*_i_* was the retention time of a certain unknown volatile compound to be measured, RT*_n_* < RT*_i_* < RT*_n_*_+1_, and the subscripts of *n* and *n* + 1 were the carbon-atom numbers of *n*-alkanes before and after the appearance of the certain unknown volatile compound in GC/MS.

A semi-quantitative analysis was employed to quantify the peak area of the identified aldehydes, and the results were presented as the peak areas of samples with standard mass, which is obtained by dividing the measured peak area by the mass of the sample.

### 2.5. Statistical Analysis

Each measurement was accomplished in triplicate. Spearman correlation analysis was performed using SPSS 26 (IBM Corp., Armonk, NY, USA). The line and curve fittings were performed using Microsoft Excel 2016 (Microsoft Corporation, Redmond, WA, USA).

## 3. Results and Discussion

To clarify the change of aldehydes during frying, the initial characteristics of oil were firstly characterized. Due to aldehydes being mainly generated by the β-cleavage of alkoxy groups formed by the homolytic cleavage of fatty acid hydroperoxides [19], the initial fatty acid composition of each oil is presented in Table 1. As shown, 10 edible oils are found to be rich in unsaturated fatty acids with different characteristics. Specifically, the fatty acid composition of OL, SF, RS, and RB is mainly oleic acid, while that of NS, SS, CO, and SB is mainly linoleic acid, and linolenic acid is prominent in NP and PL. The polyunsaturated fatty acids (PUFA) composition of these oils increased sequentially.

Moreover, antioxidants are one of the key factors affecting the quality of frying oil. Tocopherol is the most common natural antioxidant in vegetable oils, which is crucial for frying stability and increases the nutritional value of the oils [20]. Therefore, the tocopherol composition of 10 oils was also investigated (Table 1). The total content of tocopherols in the 10 oils ranged from 16.40 mg/100 g (in OL) to 236.05 mg/100 g (in NP).

The deterioration of oil is characterized by CV. To study the change of CV with time during frying, the initial CV of the 10 oils was determined (Table 1), which ranged between 2.36 and 6.30 μmol/g, indicating the initial quality of the tested 10 oils was different. Consequently, it is speculated that the diversity of the initial status of these 10 oils will have a great impact on aldehydes generated during the process of frying.

### 3.1. Effect of Frying on Aldehydes Detected in Oil

The differences between the peak areas of the aldehydes detected before and after heating for 25 h are firstly studied (Table 2), where a total of 21 aldehydes were identified in 10 oils during the whole heating process. The effect of frying on aldehydes was revealed by comparing the peak areas detected at the end of frying (25 h) with that before frying (0 h). The results showed that the peak areas and species of aldehydes changed significantly with frying: the aldehydes detected in OL, SF, RS, RB, and NS containing large amounts of oleic acid (Table 1) showed an increasing tendency; while the peak area of some aldehydes (for example, (*E*)-hex-2-enal detected in SS, 2-methylbutanal detected in CO and SB, hexanal detected in NP, and butanal detected in PL) was less than that of the initial peak areas, indicating that these aldehydes may be lost through reaction or escape during frying. The reduction in the species and peak areas of aldehydes in the roasted oils (SS and PL) was greater than that of the other oils during frying. Although some aldehydes are produced during roasting and exist in large quantities in the roasted oil, giving it a rich flavor, they are lost once fried. There are few studies on direct frying with roasting oil for reference, therefore, the specific reasons need to be further explored.

The total peak area of aldehydes detected in the 10 oils increased significantly after frying, and the increasing order was OL, SS, RB, SF, NS, RS, CO, SB, PL, and NP. For the increase in the total peak area of aldehydes, the increase in OL and SF was greater than that of SB and NP, which may be related to the content of tocopherol and unsaturated double bonds. Tocopherol may act as an antioxidant that can inhibit the formation of peroxy radicals and the subsequent formation of aldehydes, thus the more tocopherol content in oil, the fewer aldehydes are generated. In addition, the number of unsaturated bonds seems to be an important factor affecting the formation and accumulation of aldehydes. The more unsaturated bonds in oil, the fewer aldehydes generated during frying. Takhar, et al. [8] found that antioxidants could inhibit the formation of aldehydes in the oils with more double bonds, while they may promote the formation of aldehydes in the oils with fewer double bonds. OL, RB, and SF contained fewer double bands, and a large number of aldehydes have been detected in these oils, which is in agreement with the above conclusions.

After frying, the total peak area of aldehydes increased most in OL, mainly because of the increase in hexanal (55.25%). Similarly, the increase in the total peak area of aldehydes was mainly caused by the increase in hexanal in SF (55.47%), RS (46.49%), NS (58.64%), CO (57.41%), and SB (52.20%), indicating that hexanal was one of the main products that appeared in the frying of these oils. In contrast, the peak areas of hexanal in NP and PL were decreased. This difference in hexanal changes in oils is related to the content of linoleic acid, as hexanal is mainly derived from the thermal oxidation of linoleic acid [22]. The PUFA of OL, SF, RS, NS, CO, and SB was mainly linoleic acid, and the continuous decomposition of linoleic acid during frying leads to the continuous increase of hexanal. While in oils dominated by linolenic acid (NP and PL), the decomposition of linolenic acid and linoleic acid was competitive, and the decomposition rate of the former was faster than the latter, which leads to a slower increase in the formation of hexanal. With the influence of other reactions, hexanal was not accumulated but tends to lose. The increase in the total peak areas of aldehydes detected in RB and SS was mainly caused by the increases in butanal of 56.28% and 58.62%, respectively, which is a thermal oxidation product of linolenic acid [23]. The main contribution to the increase in the total peak area of aldehydes in NP was (2*E*,4*E*)-hepta-2,4-dienal (52.33%), followed by pentanal, which was mainly responsible for the increase in the total peak area of aldehydes in PL.

In addition to the changes in peak areas, there were also differences between the species of aldehydes detected before and after heating. A typical Maillard reaction product, 5-methylfuran-2-carbaldehyde, was only detected in SS and PL before frying, but not after frying, which indicates that it was unstable at frying conditions of 180 °C. Some newly-generated aldehydes were also detected after frying: (*E*)-2-methylbut-2-enal, derived from linolenic acid [24] and only detected in PL because of its high linolenic acid content; (*E*)-non-2-enal and (2*E*,4*E*)-deca-2,4-dienal, derived from linoleic acid [25]; octanal, (*E*)-dec-2-enal, and (2*E*,4*E*)-undeca-2,4-dienal, derived from oleic acid [26,27], these aldehydes were all absent before frying but occurred after frying, suggesting that their formation may require a greater amount of energy. In addition, it was found that octanal, (*E*)-dec-2-enal, and (2*E*,4*E*)-undeca-2,4-dienal were detected mainly in oils with a high oleic acid content, and their changes during frying were proportional to the oleic acid content of oil. As a result, aldehydes showed different behaviors during frying in different edible oils, and the initial composition of the oil had a great impact on them.

### 3.2. The Change in the Common Aldehydes during Frying

**Figure 1:**
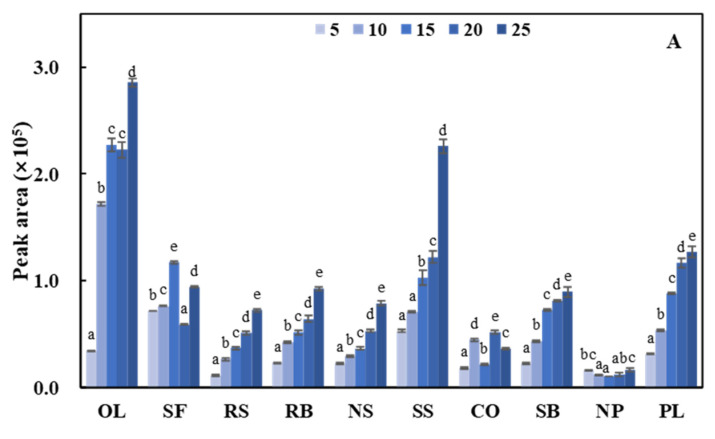

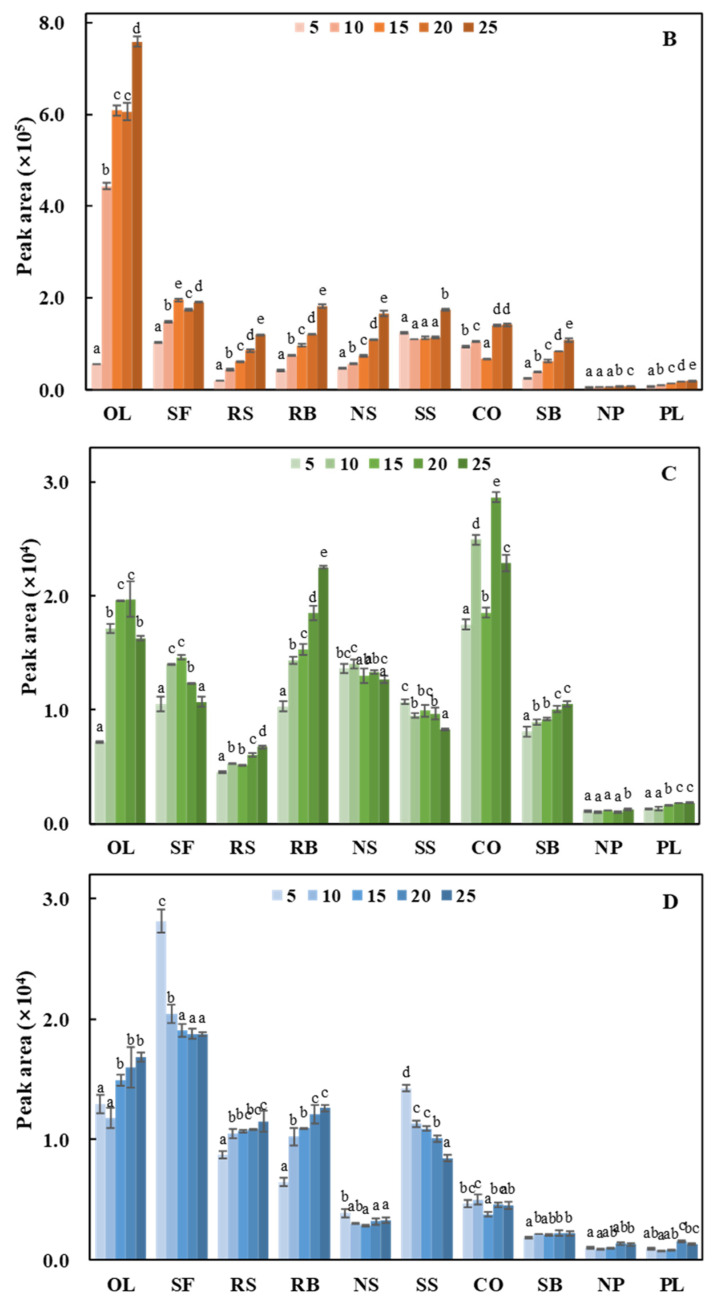
Variation in the peak area of the four volatile aldehydes common to all 10 oils with heating times from 5 to 25 h. (**A**) pentanal; (**B**) hexanal; (**C**) (*E*)-hept-2-enal; (**D**) nonanal. The different lowercase letters mean that the variance of the same oil at different heating times is significant (*p* < 0.05).

There were four aldehydes detected in all 10 oils: pentanal, hexanal, (*E*)-hept-2-enal, and nonanal, which were considered to be common. Peng, et al. [28] have also detected these four common aldehydes when deep-frying potatoes in palm, rapeseed, sunflower, and soybean oils. The patterns of change in the peak areas with the increasing heating time (5 to 25 h) for these four aldehydes are shown in Figure 1.

Pentanal is generated by the oxidation of linoleic acid [29]. During the heating process, the peak areas of pentanal in 10 oils changed significantly, either gradually increasing or fluctuating (Figure 1A). Katragadda, et al. [30] also found that pentanal fluctuated in coconut, safflower, canola, and extra virgin olive oils during heating for 6 h. Also, this result is consistent with the previously published by Ben Hammouda, et al. [31], where pentanal either increased gradually (for pure olive pomace oil) or fluctuated (for the mixture of olive pomace oil and palm oil) during 60 consecutive deep fryings. The fluctuation of pentanal may be related to its low boiling point (103 °C). Under the frying condition of 180 °C, pentanal is constantly generated due to the continuous decomposition of linoleic acid and gradually volatilizes during the intense frying process. When the generation is greater than volatilization, pentanal will gradually accumulate, as shown by the increase in the peak area of pentanal detected; conversely, the peak area of pentanal will fluctuate.

The increase in the peak area of hexanal during frying was the largest among the four common aldehydes (Figure 1B), which was mainly due to the diversity of its formation pathways. In addition to being mainly generated from linoleic acid, hexanal can also be formed from oleic acid and arachidonic acid, as well as other unsaturated aldehydes such as 2,4-decadienal [32]. As a result, the peak area of hexanal increased significantly in all oils during frying. It was observed that the peak area of hexanal detected in OL was the largest and increased the fastest, followed by SF. On the contrary, the peak area of hexanal detected in NP and PL was the smallest, which was roughly proportional to the results of the sum of oleic acid and linoleic acid in Table 1.

(*E*)-Hept-2-enal was the only unsaturated aldehyde among the four common aldehydes. It was the β-homolysis product of linoleic acid-12-hydroperoxide and can also be formed by the decomposition of 2,4-decadienal [7]. Therefore, the peak area of (*E*)-hept-2-enal was the largest in oils with more linoleic acid and (2*E*,4*E*)-deca-2,4-dienal (CO and RB). Figure 1C shows that (*E*)-hept-2-enal exhibited different trends in the 10 oils during frying: in OL and SF, its peak area increased rapidly during the early stages of heating and then decreased slightly after reaching its highest level. However, in RS, RB, SB, NP, and PL, it increased steadily over time, while it decreased slightly in NS and SS. It fluctuated greatly in CO. This variation of (*E*)-hept-2-enal among the 10 oils was probably caused by its participation in various subsequent reactions. Overall, the content of (*E*)-hept-2-enal was affected to a different extent by other components in the 10 oils, resulting in significant differences in its reactivity.

Nonanal is a thermal decomposition product derived from oleic acid [7], thus the peak area detected in oils rich in oleic acid was larger (Figure 1D). It was found that the peak area of nonanal in OL, RS, and RB gradually increased, indicating that this compound can continuously accumulate in these oils during frying. In contrast, the peak area of nonanal in SF and SS gradually decreased during frying, probably because its accumulation rate was lower than that of participating in reactions. Since the boiling point (191 °C) of nonanal is higher than the frying temperature, it is not easily volatile during frying. On the contrary, nonanal with a long carbon chain tends to react further under intense frying, causing losses. In the remaining five oils, the peak area of nonanal remained almost constant, which indicates that the accumulation rate was equal to the loss rate.

Under long-term high-temperature conditions, some of the aldehydes formed can be lost from the frying system through volatilization with steam or oil fumes, or absorbed by the fried food through mass transfer, which gives the fried food a unique flavor. In addition, some aldehydes can also dissolve in the frying oil and then react or volatilize in the subsequent frying operations [33]. These aldehydes that can accumulate and be detected in oil should be focused on, as it is they that form the basis of quality evaluation indices for frying oil. These aldehydes can characterize and affect the quality of frying oil, lead to its gradual deterioration, and also be transferred into fried foods and affect their quality. Therefore, knowledge of these aldehydes which accumulate in oil is essential for understanding the deterioration mechanism of edible oil, measuring the quality of frying oils, and for ensuring the safety of fried food. Pentanal and hexanal have usually been used as representative volatile compounds in many studies on volatile compounds [34,35], which may be related to their high accumulation.

### 3.3. Selecting Aldehydes to Evaluate the Quality of Frying Oil

During repeated frying processes, the quality of the edible oil gradually deteriorates, as indicated by the CV gradually increasing with heating time (Table S1). The correlation of the peak areas of total aldehydes and four common aldehydes with the CV of the 10 oils during frying was analyzed to evaluate their contribution to CV (Table 3). The correlation coefficients were mostly positive. The higher the correlation coefficient between the peak area of aldehydes with CV, the more the aldehydes can reflect the degree of oil deterioration, so that the peak area of the aldehydes was considered to be available for evaluating oil deterioration. After comparing, the peak area of pentanal had the most significant correlation with CV, with the highest correlation coefficients (0.543–1.000).

In the present study, pentanal, as a common aldehyde, increased significantly during frying, which can be easily and continuously measured, and it is highly correlated with CV, therefore, it could be used as an evaluation index of oil deterioration during frying and has universality for all edible oils. The increase of the peak area of pentanal was almost linear with the increase of CV in the 10 oils, which was expressed by the linear fitting model (Figure S1). Table 4 shows the fitting results by using Equation (2) for 0 ≤ *t* ≤ 25:

(2) $${\mathrm{CV}}_{t}-{\mathrm{CV}}_{0}=k\left({\mathrm{Sp}}_{t}-{\;\mathrm{Sp}}_{0}\right),$$

where CV*_t_* and CV_0_ refer to the carbonyl values at times, *t*, and 0 (before frying), respectively; the slope, *k*, indirectly reflects the increase in CV with the increase in the peak area of pentanal during frying; and Sp*_t_* and Sp_0_ refer to the peak area of pentanal at times, *t*, and 0 (before frying), respectively. During the frying process, CV increases gradually with the increase of the peak area of pentanal, reflecting the deeper deterioration of the oil.

The value of *k* for NP was the highest of the 10 oils at 24.91 × 10^−4^, while that of OL was the lowest at 1.51 × 10^−4^, a 16-fold difference. To explore the reasons for this significant variation in *k* value between the 10 oils, *k* was linked to the initial composition of the oils, which was the source of any change occurring in the oil. Both the fatty acid and tocopherol compositions of oils can influence the CV and the formation of aldehydes during frying. Unsaturated fatty acids, especially linoleic acid, are responsible for a high CV and the production of pentanal [29], while tocopherol is the main antioxidant in oil, which can slow down its oxidation and thus reduce CV and the production of pentanal. Therefore, it is reasonable to consider the simultaneous influence of unsaturated fatty acids and tocopherol when investigating the relationship between *k* and the initial composition of the oil. Palmitic acid is relatively stable during the process of oil oxidation, and the ratio of unsaturated fatty acids to palmitic acid is usually used to indicate the degree of oil deterioration [36]. Therefore, the introduction of palmitic acid also needs to be considered when investigating the effect of unsaturated fatty acids.

**Figure 2:**
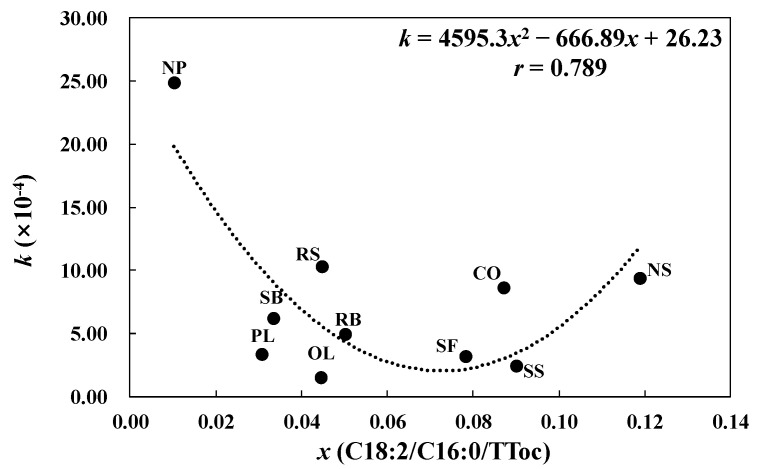
Relationship between *k* and the initial composition of oil during frying. *k* is the slope of the change in carbonyl value with the change in the peak area of pentanal and *x* represents the ratio of the initial contents of linoleic acid to that of palmitic acid and total tocopherols.

After correlating the value of *k* in Equation (2) with the initial composition of unsaturated fatty acids and tocopherol in the 10 oils (Table 1) and comparing the correlation results, the best fitting relationship was found between *k* and the initial content ratio of linoleic acid (C18:2) to palmitic acid (C16:0) and total tocopherols (TToc). Equation (3) shows how the data on *k* and the ratio of the initial contents of C18:2 to C16:0 and TToc can be fitted, which gives a determination coefficient of 0.789 with the data plotted in Figure 2.

(3) $$k=4595.3{\left(\frac{C18:2}{C16:0\times \mathrm{TToc}}\right)}^{2}-666.89\left(\frac{C18:2}{C16:0\times \mathrm{TToc}}\right)+26.23,$$

Equation (4) can then be obtained by substituting Equation (3) into Equation (2) and rearranging. This is valid for heating times between 0 and 25 h.

(4) $${\mathrm{CV}}_{t}=\left[4595.3{\left(\frac{C18:2}{C16:0\times \mathrm{TToc}}\right)}^{2}-666.89\left(\frac{C18:2}{C16:0\times \mathrm{TToc}}\right)+26.23\right]\left({\mathrm{Sp}}_{t}-{\mathrm{Sp}}_{0}\right){+\mathrm{CV}}_{0},$$

From Equation (4), the CV of frying oil can be predicted by using the initial content ratios and changes in the peak area of pentanal, thus enabling the quality of frying oil to be evaluated using high-precision mass spectrometry without pretreatment instead of using complex tests based on chemical reagents for determining the CV.

This model has universal applicability because it combines the initial composition and deterioration characteristics of 10 prevalent edible oils, which have a wide range of fatty acid composition and tocopherol content, and the initial quality is also taken into account. However, it should be noted that the model has some limitations, mainly reflected in two aspects: (1) In addition to the common tocopherol, there are other antioxidants or pro-oxidants in edible oil, which may play a role in the stability of the oil during frying, thus affecting the use of the model; (2) Different frying foods will have an impact on the deterioration of the oil during frying due to the huge variation in composition. Therefore, the model applies to the prediction of edible oil quality in the process of long-term intermittent frying of French fries. It needs to be properly adjusted and improved when used for other frying foods or frying processes.

## 4. Conclusions

In this work, the aldehydes were determined in 10 oils during frying using HS-GC/MS. The effect of frying on aldehydes was revealed by comparing the peak areas of 21 aldehydes before and after frying. Subsequently, by analyzing the increase in the peak areas of the four common aldehydes (pentanal, hexanal, (*E*)-hept-2-enal, and nonanal) with time and their positive correlations with CV, pentanal was determined as the index for evaluating the quality of frying oils. As a result, a prediction model was established to predict the CV of frying oil by using the ratio of the initial contents of C18:2 to C16:0 and TToc of the edible oil combined with the peak area of pentanal during frying. The model has a wide range of prospective applications, which demonstrates the feasibility of using volatile compounds as a quality evaluation index for frying oil. Subsequent research should focus on further improving the applicability of this model to different frying systems.

## Figures and Tables

**Table 1:** Fatty acid and tocopherol compositions, and carbonyl values of samples of 10 types of oil before frying. Reprinted/adapted with permission from Ref. [21]. Copyright 2020, Elsevier.

Name	Fatty Acid (%)							Tocopherol (mg/100 g of Oil)					Carbonyl Value (μmol/g)
	C16:0	C18:0	C18:1	C18:2	C18:3	PUFA	C18:1 + C18:2	α	β	γ	δ	TToc	
OL	8.58 ± 0.14	2.44 ± 0.03	81.51 ± 0.15	6.27 ± 0.13	0.35 ± 0.02	6.62	87.78	14.44 ± 0.94	0.57 ± 0.03	1.40 ± 0.04	-	16.40	3.41 ± 0.21
SF	4.31 ± 0.13	1.71 ± 0.17	79.69 ± 0.80	13.58 ± 0.60	0.33 ± 0.23	13.91	93.27	38.67 ± 0.63	0.70 ± 0.01	0.90 ± 0.01	-	40.27	4.67 ± 0.32
RS	3.22 ± 0.08	1.28 ± 0.02	67.71 ± 0.77	18.31 ± 0.30	9.26 ± 0.58	27.57	86.02	46.30 ± 0.39	0.50 ± 0.03	74.66 ± 1.56	5.46 ± 0.08	126.92	4.32 ± 0.29
RB	14.98 ± 0.10	1.53 ± 0.14	46.68 ± 0.14	35.34 ± 0.55	1.47 ± 0.31	36.81	82.02	38.37 ± 1.08	2.10 ± 0.15	6.13 ± 0.16	0.35 ± 0.02	46.95	5.92 ± 0.38
NS	8.12 ± 0.08	4.71 ± 0.02	41.25 ± 0.14	45.67 ± 0.25	0.25 ± 0.07	45.92	86.92	-	-	47.32 ± 0.92	-	47.32	2.36 ± 0.11
SS	8.20 ± 0.16	4.57 ± 0.06	38.88 ± 0.20	47.74 ± 0.37	0.21 ± 0.02	47.95	86.62	-	-	64.55 ± 4.15	-	64.55	3.02 ± 0.24
CO	10.67 ± 0.21	1.47 ± 0.01	28.49 ± 0.22	58.35 ± 0.56	-	58.35	86.84	17.00 ± 0.24	0.47 ± 0.00	44.17 ± 1.06	1.18 ± 0.01	62.81	4.48 ± 0.40
SB	10.09 ± 0.11	3.24 ± 0.15	19.21 ± 0.32	57.83 ± 0.35	9.63 ± 0.21	67.46	77.04	27.20 ± 0.56	1.97 ± 0.27	123.31 ± 4.25	18.89 ± 0.51	171.37	6.30 ± 0.13
NP	5.16 ± 0.01	1.31 ± 0.03	16.68 ± 0.07	12.59 ± 0.04	64.26 ± 0.04	76.85	29.27	1.86 ± 0.11	-	74.75 ± 0.84	159.44 ± 0.80	236.05	2.46 ± 0.17
PL	5.10 ± 0.02	1.54 ± 0.03	15.18 ± 0.03	12.34 ± 0.03	65.83 ± 0.03	78.17	27.52	2.37 ± 0.13	1.32 ± 0.04	73.78 ± 1.15	1.04 ± 0.07	78.51	3.12 ± 0.11

Abbreviations: C16:0, palmitic acid; C18:0, stearic acid; C18:1, oleic acid; C18:2, linoleic acid; C18:3, linolenic acid; PUFA, polyunsaturated fatty acids; C18:1 + C18:2, the content sum of oleic acid and linoleic acid; TToc, total tocopherols; OL, olive oil; SF, safflower oil; RS, rapeseed oil; RB, rice bran oil; NS, natural sesame oil; SS, sesame oil; CO, corn oil; SB, soybean oil; NP, natural perilla oil; PL, perilla oil; and “-”, not detected.

**Table 2:** Difference between the peak areas of 21 volatile aldehydes detected in 10 edible oils before and after heating for 25 h.

No.	Volatile Compound	RI	RIr	Difference between the Peak Areas of Volatile Aldehydes before and after Heating for 25 h (×10^3^)									
				OL	SF	RS	RB	NS	SS	CO	SB	NP	PL
1	Butanal	619	601	* 219.41 ± 6.65	-	0.57 ± 0.01	425.03 ± 2.36	2.53 ± 0.13	473.79 ± 16.34	-	−12.92 ± 0.77	-	−71.43 ± 4.04
				^#^ (16.08)	-	(0.28)	(56.28)	(1.07)	(58.62)	-	(7.56)	-	(50.73)
2	(*E*)-But-2-enal	692	657	8.65 ± 0.38	-	7.55 ± 0.52	-	-	-	-	-	11.54 ± 0.70	52.06 ± 0.86
				(0.63)	-	(3.68)	-	-	-	-	-	(17.45)	(36.98)
3	2-Methylbutanal	704	664	-	4.21 ± 0.29	-	6.84 ± 0.25	10.03 ± 0.42	8.35 ± 0.33	−1.45 ± 0.07	−8.87 ± 0.30	-	6.29 ± 0.30
				-	(1.28)	-	(0.91)	(4.24)	(1.03)	(0.72)	(5.19)	-	(4.46)
4	Pentanal	724	701	275.20 ± 4.18	86.27 ± 1.79	63.73 ± 1.53	84.66 ± 1.61	68.03 ± 1.25	202.76 ± 6.15	31.13 ± 0.78	72.38 ± 3.96	14.85 ± 1.71	126.90 ± 5.45
				(20.16)	(26.19)	(31.03)	(11.21)	(28.80)	(25.09)	(15.38)	(42.34)	(22.44)	(90.13)
5	(*E*)-2-Methylbut-2-enal	766	745	-	-	-	-	-	-	-	-	-	4.43 ± 0.15
				-	-	-	-	-	-	-	-	-	(3.14)
6	(*E*)-Pent-2-enal	779	759	7.19 ± 0.21	-	3.87 ± 0.30	-	-	-	-	-	6.03 ± 0.08	14.94 ± 0.46
				(0.53)	-	(1.88)	-	-	-	-	-	(9.11)	(10.61)
7	Hexanal	824	802	754.02 ± 11.37	182.71 ± 2.28	95.47 ± 0.95	170.96 ± 5.27	138.54 ± 6.35	136.15 ± 1.43	116.24 ± 2.97	89.24 ± 4.69	−3.43 ± 0.42	−5.27 ± 0.31
				(55.25)	(55.47)	(46.49)	(22.64)	(58.64)	(16.84)	(57.41)	(52.20)	(5.18)	(3.74)
8	Furan-2-carbaldehyde	854	830	-	-	-	-	-	−20.35 ± 0.27	-	-	-	-
				-	-	-	-	-	(2.52)	-	-	-	-
9	(*E*)-Hex-2-enal	877	864	2.31 ± 0.09	2.17 ± 0.15	-	2.83 ± 0.20	0.71 ± 0.01	−6.04 ± 0.19	3.76 ± 0.25	0.67 ± 0.02	-	−10.78 ± 0.55
				(0.17)	(0.66)	-	(0.37)	(0.30)	(0.75)	(1.85)	(0.39)	-	(7.66)
10	Heptanal	927	903	27.14 ± 1.02	7.57 ± 0.26	6.86 ± 0.46	5.82 ± 0.28	1.75 ± 0.05	4.72 ± 0.06	3.41 ± 0.07	1.99 ± 0.12	-	−5.96 ± 0.29
				(1.99)	(2.30)	(3.34)	(0.77)	(0.74)	(0.58)	(1.68)	(1.16)	-	(4.23)
11	(*E*)-Hept-2-enal	981	956	16.29 ± 0.18	10.42 ± 0.57	4.5 ± 0.07	20.51 ± 0.45	6.38 ± 0.03	7.03 ± 0.23	22.86 ± 0.73	7.75 ± 0.13	1.27 ± 0.07	1.82 ± 0.05
				(1.19)	(3.16)	(2.19)	(2.72)	(2.70)	(0.87)	(11.29)	(4.53)	(1.92)	(1.29)
12	5-Methylfuran-2-carbaldehyde	984	980	-	-	-	-	-	−13.4 ± 0.62	-	-	-	−8.23 ± 0.43
				-	-	-	-	-	(1.66)	-	-	-	(5.84)
13	Octanal	1029	1003	24.15 ± 0.92	6.53 ± 0.45	3.09 ± 0.17	2.66 ± 0.12	1.59 ± 0.10	3.95 ± 0.09	1.11 ± 0.01	-	-	-
				(1.77)	(1.98)	(1.50)	(0.35)	(0.67)	(0.49)	(0.55)	-	-	-
14	(2*E*,4*E*)-Hepta-2,4-dienal	1036	1015	2.03 ± 0.08	-	5.64 ± 0.38	3.20 ± 0.16	-	-	-	8.95 ± 0.13	34.62 ± 0.85	35.25 ± 1.51
				(0.15)	-	(2.75)	(0.42)	-	-	-	(5.23)	(52.33)	(25.03)
15	(*E*)-Oct-2-enal	1083	1064	4.77 ± 0.30	1.39 ± 0.03	1.07 ± 0.00	3.36 ± 0.18	0.62 ± 0.02	2.03 ± 0.07	2.29 ± 0.09	1.68 ± 0.06	-	-
				(0.35)	(0.42)	(0.52)	(0.45)	(0.26)	(0.25)	(1.13)	(0.98)	-	-
16	Nonanal	1132	1104	16.85 ± 0.39	18.73 ± 0.15	9.31 ± 0.70	11.54 ± 0.53	0.56 ± 0.12	6.94 ± 0.06	4.56 ± 0.30	1.60 ± 0.04	1.28 ± 0.09	0.77 ± 0.00
				(1.23)	(5.69)	(4.53)	(1.53)	(0.24)	(0.86)	(2.25)	(0.94)	(1.94)	(0.55)
17	(*E*)-Non-2-enal	1187	1165	1.49 ± 0.05	0.80 ± 0.02	-	-	-	2.34 ± 0.12	-	-	-	-
				(0.11)	(0.24)	-	-	-	(0.29)	-	-	-	-
18	(*E*)-Dec-2-enal	1294	1263	3.55 ± 0.19	3.46 ± 0.26	1.38 ± 0.05	2.16 ± 0.09	0.92 ± 0.03	-	-	-	-	-
				(0.26)	(1.05)	(0.67)	(0.29)	(0.39)	-	-	-	-	-
19	(2*E*,4*E*)-Deca-2,4-dienal	1327	1316	-	-	-	2.63 ± 0.04	-	-	3.45 ± 0.01	1.66 ± 0.04	-	-
				-	-	-	(0.35)	-	-	(1.70)	(0.97)	-	-
20	(*E*)-Undec-2-enal	1350	1360	-	3.20 ± 0.02	2.34 ± 0.15	11.87 ± 0.50	4.60 ± 0.25	-	15.12 ± 1.45	6.85 ± 0.26	-	-
				-	(0.97)	(1.14)	(1.57)	(1.95)	-	(7.47)	(4.00)	-	-
21	(2*E*,4*E*)-Undeca-2,4-dienal	1396	1420	1.72 ± 0.12	1.91 ± 0.10	-	1.08 ± 0.06	-	-	-	-	-	-
				(0.13)	(0.58)	-	(0.14)	-	-	-	-	-	-
	Total			1364.76	329.37	205.37	755.15	236.25	808.27	202.48	170.97	66.16	140.81

The aldehydes were identified by comparison of the mass spectrum and the retention index (RI) with those in the NIST 17 Mass Spectral Library and similar phase columns used for the study of frying oil. RIr means the RI in references. “*”—Difference between peak areas before and after heating for 25 h (×10^3^); “^#^”—The percentage given in parenthesis is the change in peak area, denoted by *, as a percentage of the total aldehydes peak area difference given in the last row (%); “-”—Not detected.

**Table 3:** Correlation between the peak area of total aldehydes and common aldehydes with the carbonyl value (CV) of 10 types of oil during frying.

Name	Correlation Coefficient with CV				
	Total Aldehydes	Pentanal	Hexanal	(*E*)-Hept-2-enal	Nonanal
OL	1.000 **	0.943 **	0.943 **	0.657	0.943 **
SF	0.829 *	0.543	0.829 *	0.486	−0.143
RS	1.000 **	1.000 **	0.943 **	0.943 **	1.000 **
RB	1.000 **	1.000 **	1.000 **	1.000 **	1.000 **
NS	1.000 **	1.000 **	1.000 **	−0.029	0.371
SS	0.371	1.000 **	0.657	0.029	−0.143
CO	0.771	0.714	0.829 *	0.714	0.086
SB	1.000 **	1.000 **	1.000 **	1.000 **	0.943 **
NP	0.029	0.600	−0.029	0.714	0.771
PL	1.000 **	1.000 **	0.143	0.943 **	0.771

Significance: *, *p* ≤ 0.05; **, *p* ≤ 0.01.

**Table 4:** Linear fitting results of change in the peak area of pentanal (Sp) with change in carbonyl value (CV) of 10 types of oil during frying with *k* calculated using Equation (2).

Name	*k* (×10^−4^)	*R* ^2^
NP	24.91	0.316
RS	10.32	0.803
NS	9.42	0.884
CO	8.63	0.388
SB	6.20	0.927
RB	4.98	0.977
PL	3.33	0.989
SF	3.23	0.444
SS	2.44	0.844
OL	1.51	0.937

## Data Availability

Data are contained within the article.

## Supplementary Materials

The following supporting information can be downloaded at: https://www.mdpi.com/article/10.3390/foods11162413/s1, Figure S1: Relationship between the change in the peak areas of pentanal (Sp) and the change in carbonyl value (CV) during frying; Table S1: The carbonyl value of 10 types of oil during frying.
