# Supplementary material for: An Evaluation Model for the Quality of Frying Oil Using Key Aldehyde Detected by HS-GC/MS
Source: Foods. 2022 Aug 11;11(16):2413. doi: 10.3390/foods11162413 (PMC9407462; doi:10.3390/foods11162413)
Supplement: Supplementary file 1 [file foods-11-02413-s001.zip › foods-1841365-supplementary.pdf]

Supplementary information

An Evaluation Model for the Quality of Frying Oil Using Key Aldehyde Detected by HS-GC/MS

Xiaofang Liu [1,2,3], Shuo Wang [1,4], Shigeru Tamogami [1], Jieyu Chen [1] and Han Zhang [1,*]

Table S1. The carbonyl value of 10 types of oil during frying.

Heating time (h)	Carbonyl value (μmol/g)									
	Olive	Safflower	Rapeseed	Rice bran	Natural sesame	Sesame	Corn	Soybean	Natural perilla	Perilla
5	11.23 ± 0.40	14.70 ± 0.75	16.88 ± 0.30	12.53 ± 0.62	12.15 ± 0.69	12.59 ± 0.94	11.65 ± 1.26	17.32 ± 0.86	11.43 ± 0.16	9.74 ± 0.55
10	22.59 ± 1.82	21.97 ± 0.78	33.15 ± 2.73	21.28 ± 1.77	23.58 ± 1.60	20.96 ± 2.05	21.93 ± 1.51	26.35 ± 1.64	23.47 ± 1.55	20.47 ± 1.01
15	31.50 ± 0.65	31.82 ± 1.64	43.52 ± 2.62	29.17 ± 1.04	37.90 ± 1.05	29.64 ± 2.85	34.73 ± 1.23	36.11 ± 0.83	34.75 ± 1.00	31.69 ± 1.54
20	38.59 ± 0.99	37.64 ± 1.20	52.69 ± 0.87	37.78 ± 2.87	48.20 ± 1.02	33.45 ± 1.96	40.45 ± 0.17	43.58 ± 3.94	43.40 ± 2.64	42.04 ± 2.43
25	48.72 ± 1.62	43.99 ± 3.76	59.28 ± 1.47	45.77 ± 3.13	57.46 ± 0.72	45.39 ± 3.88	52.34 ± 1.72	54.67 ± 2.82	52.67 ± 3.47	46.68 ± 0.94

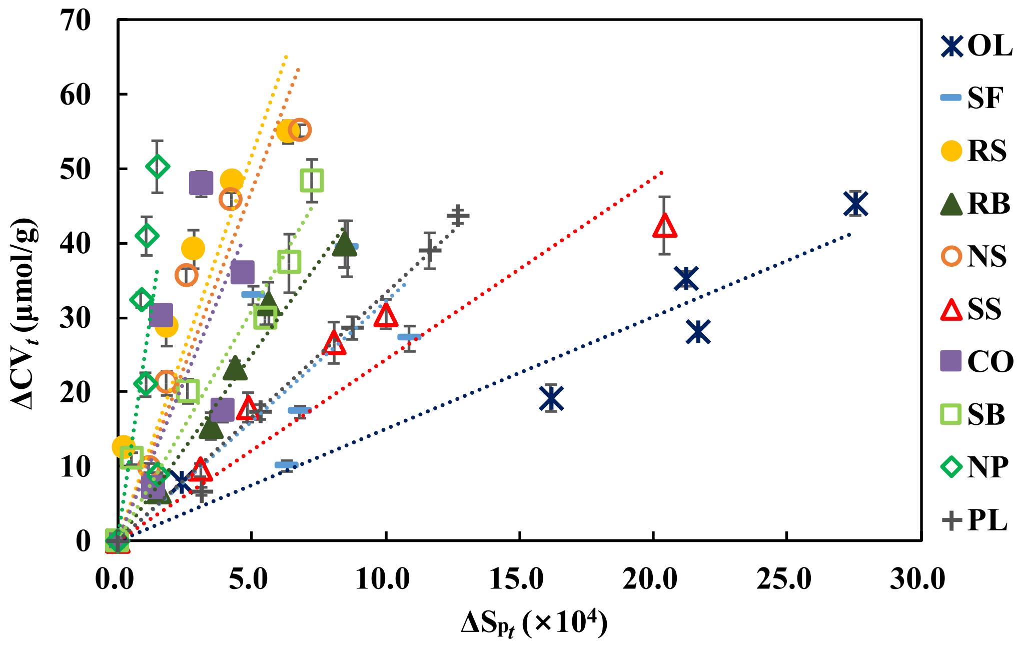

Figure S1. Relationship between the change in the peak areas of pentanal (Sp) and the change in carbonyl value (CV) during frying. Abbreviations: OL, olive oil; SF, safflower oil; RS, rapeseed oil; RB, rice bran oil; NS, natural sesame oil; SS, sesame oil; CO, corn oil; SB, soybean oil; NP, natural perilla oil; PL, perilla oil.
